# Nanoparticle-Mediated Targeted Drug Delivery Systems

**DOI:** 10.3390/pharmaceutics17111480

**Published:** 2025-11-17

**Authors:** Toshihiko Tashima, Nicolas Tournier

**Affiliations:** 1 Tashima Laboratories of Arts and Sciences, 1239-5 Toriyama-cho, Kohoku-ku, Yokohama 222-0035, Japan; 2 Laboratoire d’Imagerie Biomédicale Multimodale, BIOMAPS, Université Paris-Saclay, CEA, CNRS, Inserm, Service Hospitalier Frédéric Joliot, 4 Place du Général Leclerc, 91401 Orsay, France

We will be serving as the Guest Editors for this very interesting Special Issue on “Nanoparticle-Mediated Targeted Drug Delivery Systems”. In drug discovery and development, selective and efficient transmembrane delivery of therapeutic substances into cells remains a major challenge. This difficulty arises from several factors, including molecular size, hydrophilic/hydrophobic balance, and the substrate specificity of membrane transporters—such as influx transporters from the solute carrier (SLC) family [1,2] and efflux transporters like multidrug resistance protein 1 (MDR1, also known as P-glycoprotein) [3,4]—as well as limited enzymatic stability. These barriers are rooted in complex transport and metabolic systems within the cell, based on the machinery systems regulated by structuralism, wherein the structure of biological components fundamentally determines their function and interaction within cellular pathways [5,6]. As a result, numerous pharmaceutical challenges arise. Substance impermeability significantly contributes to low bioavailability, particularly in the case of central nervous system (CNS) drugs, whose entry from the bloodstream into the brain is restricted by the blood–brain barrier (BBB) [7,8]. This barrier is maintained by tight junctions and efflux transporters such as MDR1. In addition, off-target side effects may occur due to improper tissue distribution or unintended drug–protein interactions during systemic distribution. Substances such as RNAs and peptides are often enzymatically degraded in the serum or small intestine before reaching their target sites. Therefore, innovative drug delivery systems are needed to overcome these challenges. Nanodelivery systems have shown promise in addressing these issues [9]. Notably, microRNAs (miRNAs) encapsulated in exosomes released from donor cells can be delivered to recipient cells through intercellular communication, without degradation by RNases [10,11]. Exosomes are small, lipid-based extracellular vesicles. Interestingly, cancer cells have exploited this system by utilizing exosomal miRNAs to modulate the tumor microenvironment, particularly during metastasis. Similarly, nanoparticles can encapsulate therapeutic agents as cargo to protect them from degradation and unwanted interactions. Comirnaty^®^ (tozinameran, BNT162b2), approved by the FDA on 23 August 2021, and Spikevax^®^ (Moderna COVID-19 vaccine), approved on 31 January 2022, are examples of lipid nanoparticle-based mRNA vaccines developed for COVID-19 treatment [12]. Furthermore, nanoparticles can be functionalized with targeting vectors that enhance cellular uptake, such as monoclonal antibodies (mAbs) [13], cell-penetrating peptides (CPPs) [14], or tumor-homing peptides (THPs) [15]. Although the mechanisms of CPP internalization are not fully understood, both endocytosis and direct translocation are generally accepted pathways. It is thought that negatively charged heparan sulfate chains, which extend from proteoglycans (HSPGs) on the cell surface, facilitate receptor-mediated endocytosis by serving as receptors for cationic CPPs, such as the trans-activator of transcription (TAT) peptide (GRKKRRQRRRPQ) derived from HIV-1 [16]. Among representative THPs, RGD peptides (Arg-Gly-Asp) specifically bind to αvβ3 and αvβ5 integrins on the surface of cancer cells [17], while NGR peptides (Asn-Gly-Arg) target the receptor aminopeptidase N [18]. Although the binding of THPs to their receptors can induce various biological responses, endocytosis is one of the primary mechanisms involved. Additionally, the molecular Trojan horse strategy utilizes monoclonal antibodies (mAbs) targeting specific receptors to initiate receptor-mediated endocytosis [19]. For example, Izcargo^®^ (pabinafusp alfa), a conjugate of an anti-transferrin receptor (TfR) mAb and human iduronate-2-sulfatase, was approved in Japan in May 2021 for the treatment of all forms of mucopolysaccharidosis type II (MPS II). This therapeutic crosses the BBB via TfR-mediated transcytosis [20]. Numerous types of nanoparticles functionalized with such targeting vectors have since been developed [21,22]. This Special Issue aims to highlight recent advances and emerging trends in this rapidly evolving field.

Currently, nanoparticle-based drug delivery systems are widely utilized due to their exceptional versatility and adaptability, which can be achieved through appropriate material selection and practical surface modifications to suit various therapeutic needs and conditions. Nanoparticles are primarily composed of one or more of the following constituents: (i) biodegradable synthetic polymers, (ii) natural polymers (e.g., chitosan, PLGA, PGA), (iii) lipids (e.g., liposomes, micelles, exosomes), (iv) inorganic materials (e.g., gold, silica, Fe_3_O_4_), (v) organic materials (e.g., albumin, monoclonal antibodies, virosomes), (vi) emulsions, and (vii) other specialized components. Biodegradability and biocompatibility are critical factors in minimizing adverse side effects. In practice, functionalized or engineered nanoparticles [23] have been developed with various specialized features, including: (i) passive targeting based on the enhanced permeability and retention (EPR) effect, particularly in solid tumors [24,25]; (ii) active targeting through ligand–receptor interactions that facilitate receptor-mediated endocytosis, using the aforementioned vectors; (iii) magnetic responsiveness; (iv) pH sensitivity; (v) thermosensitivity; (vi) enteric coating for oral administration [26]; and (vii) other application-specific functionalities. Nanoparticles are employed in drug therapy for the treatment of a wide range of diseases, including (i) cancers; (ii) central nervous system (CNS) disorders such as Alzheimer’s disease, Parkinson’s disease, ischemic stroke, and glioma; (iii) infectious diseases, including influenza, cytomegalovirus, and COVID-19; (iv) cardiovascular diseases; (v) pulmonary diseases; (vi) ocular diseases; and (vii) other conditions. As drug carriers, nanoparticles can load or encapsulate a variety of therapeutic agents, including (i) low-molecular-weight compounds; (ii) peptides; (iii) nucleic acids (e.g., RNAs and DNAs); and (iv) other bioactive molecules.

Active targeting based on ligand–receptor interactions, which facilitate receptor-mediated endocytosis through specific vectors, has been utilized to deliver therapeutic agents to selected tissues such as (1) the heart, (2) (3) the respiratory system, and (4) the brain. While drug delivery to the brain has been extensively studied [23], research on targeted delivery to other tissues remains limited.

(1) Toshihiko Tashima has focused on nanoparticle-based delivery to the heart using nanodelivery systems. In this approach, nanoparticles actively traverse the cardiac capillary endothelium via receptor-mediated transcytosis. Conversely, during myocardial infarction (MI) and chronic heart failure (CHF), the tight junctions of the cardiac capillary endothelium become disrupted, allowing nanoparticles to passively diffuse through these gaps into the heart [27]. This passive transport mechanism resembles the EPR effect observed in solid tumors [24,25].

(2) Devesh U. Kapoor et al. [28] reviewed the use of polymeric nanoparticles for targeted lung cancer therapy. Reported delivery routes include intravenous, pulmonary (inhalation), oral, and intratumoral administration. Multifunctional nanoparticles have been shown to enhance tumor-specific accumulation and cellular uptake, thereby improving bioavailability while reducing systemic toxicity. (3) Moreover, the coronavirus disease 2019 (COVID-19) pandemic, caused by severe acute respiratory syndrome coronavirus 2 (SARS-CoV-2) [29], had a devastating impact on global public health and triggered the most significant economic crisis in over a century. Comirnaty^®^ and Spikevax^®^, both mRNA-encapsulated lipid nanoparticles, were administered via intramuscular injection; however, injection site reactions such as pain, redness, and swelling were commonly reported [30]. Nicolette Frank et al. investigated novel nasal mucoadhesive nanoformulations containing lipid-soluble epigallocatechin-3-gallate (EGCG) for the treatment of long COVID. EGCG-monopalmitate (EC16m), a derivative of green tea, is known to possess multiple antiviral mechanisms in addition to anti-inflammatory, antioxidant, and neuroprotective properties. It is hypothesized that persistent SARS-CoV-2 infection in the nasal neuroepithelium may invade support and stem cells in the olfactory mucosa, thereby contributing to neurological symptoms, including CNS inflammation and oxidative stress. A 5 min exposure to a saline-based EC16m mucoadhesive nanoformulation—specifically, formulation D (FD), with a particle size of 257 ± 134 nm and a Zeta potential of −51.31 ± 1.22 mV—resulted in 99.9% inactivation of β-coronavirus OC43 in TCID50 assays using MRC-5 cells. In contrast, when HCT-8 human intestinal epithelial cells were exposed to the same formulation for one hour, no significant difference in cell viability was observed compared to the untreated control. Typically, a full nasal spray volume is approximately 0.07 mL; each spray of the FD formulation delivers about 455 million EC16m nanoparticles, an amount considered sufficient to inactivate coronavirus present in the olfactory mucosa. Additionally, a small portion of EC16m nanoparticles is expected to enter the CNS before clearance by nasal cilia, where they subsequently release free EGCG [31].

(4) Nasal administration of nanoparticles is considered a promising strategy for the treatment of CNS diseases, offering an alternative route for brain delivery that bypasses the BBB. Despite this, non-invasive delivery across the BBB remains a major pharmacokinetic challenge in CNS drug development. Alberta De Capua et al. reported the peptide functionalization of emulsion-based nanocarriers to enhance BBB penetration. The CRT peptide (CRTIGPSVC), a bioactive iron-mimetic peptide, selectively targets bEnd.3 cells—a widely used in vitro model of the BBB—via Tf–TfR interactions. Paclitaxel-loaded nanoemulsions coated with two functional layers—chitosan and hyaluronic acid conjugated to the CRT peptide—showed a 41.5% increase in cellular uptake compared to the negative control in bEnd.3 cells. Additionally, these functionalized nanoemulsions resulted in a 33.05% increase in cytotoxicity compared to undecorated polyethylene glycol (PEG)-based nanoemulsions. Paclitaxel, a plant-derived alkaloid, inhibits mitotic progression. The CRT–PEG–streptavidin–hyaluronic acid–biotin–chitosan nanoemulsions exhibited an average diameter of 137 ± 7 nm and a Zeta potential of −30 ± 1 mV [32]. The use of TfR-mediated transcytosis represents a promising strategy for nanoparticle delivery to the brain. Streptavidin, a biotin-binding protein, enables facile surface modification of nanoparticles through strong non-covalent interactions [33,34]. Other examples of non-covalent, transient linkages include electrostatic interactions, such as those formed between glutamic acid (Glu) and arginine (Arg) residues [35].

Nanodelivery systems are suitable to deliver certain types of substances, such as polyphenols including (5) resveratrol, cytotoxic agents including (6) cabazitaxel and RAS-selective lethal 3, and enzymatically unstable materials including (7) peptides, (8) nucleic acids, and (9) antibiotics.

(5) Resveratrol (Res) is a type of polyphenol found in the skin of red grapes and other botanicals, and it possesses strong antioxidant potential. Kengo Banshoya et al. developed a water-soluble nanomicellar formulation loaded with trans-resveratrol using polyethylene glycol monostearate (stPEG) for the treatment of intracerebral hemorrhage (ICH). They also successfully formulated poorly water-soluble compounds such as α-tocopherol [36] and coenzyme Q10 [37] into water-soluble stPEG micelles. Trans-resveratrol reduces oxidative stress, which plays an important role in brain injury following ICH. In an in vivo assay using a collagenase-induced ICH mouse model, stPEG40/Res micelles administered via the tail vein exhibited neuroprotective effects by suppressing motor dysfunction, compared to stPEG40 micelles alone as a control [38]. Nanoparticles with diameters smaller than 100 nm can evade rapid sequestration by the reticuloendothelial system in the liver, spleen, and lungs [39]. The diameter of stPEG/Res micelles suitable for intravenous administration was adjusted within this range—85.4 nm for stPEG10/Res (Res content: 10.3% *w*/*w*) and 11.8 nm for stPEG40/Res (Res content: 8.6% *w*/*w*). Therefore, poorly water-soluble, low-molecular-weight compounds such as α-tocopherol, coenzyme Q10, and (4) Res can be effectively delivered using this system.

(6) Remya Valsalakumari et al. investigated the potential of cabazitaxel (CBZ) and RAS-selective lethal 3 (RSL3) in combinatorial nanomedicine. It is well established that combination therapies in cancer treatment often exhibit greater efficacy than monotherapies. In this context, the combination of CBZ and RSL3, co-encapsulated within poly(2-ethyl butyl cyanoacrylate) (PEBCA) nanoparticles, produced synergistic effects against various breast cancer cell lines [40].

(7) Nanodelivery systems are effective for delivering enzymatically unstable substances such as nucleic acids and peptides. Angiotensin-converting enzyme (ACE) inhibitors, such as captopril, were originally discovered from snake venom toxins [41]. Thus, venom-derived toxins have considerable potential as drug leads [42,43], despite the historical anecdote that Cleopatra VII, Queen of the Ptolemaic Kingdom of Egypt, is believed to have committed suicide by snakebite. As an example of venom-based substance delivery, Álisson E. F. Alves et al. reported the development of snake venom-loaded nanobiosystems for advanced medical applications [44]. Snake venoms, especially in their structurally unmodified form, exhibit diverse biological activities, including cytotoxic, neurotoxic, and haemotoxic effects. Encapsulation of these toxins in nanocarriers can not only protect them from enzymatic degradation but also help mitigate off-target side effects by controlling their biodistribution. Interestingly, certain snake venom components—such as bradykinin-potentiating peptides (BPPs) derived from *Bothrops jararaca* venom [45]—function as cell-penetrating peptides (CPPs) and can thus serve as vectors when incorporated into nanoparticles. Therefore, snake venoms possess both pharmacodynamic and pharmacokinetic utility in nanoparticle-based drug delivery systems.

(8) Furthermore, the clustered regularly interspaced short palindromic repeats (CRISPR)/CRISPR-associated (Cas) system has emerged as a revolutionary and straightforward tool for genome editing [46,47]. However, in vivo gene editing remains significantly more challenging than in vitro or ex vivo approaches, due to factors such as the enzymatic instability of RNAs, difficulty in achieving selective distribution to target tissues, and barriers to transmembrane transport into cells. In contrast, in vitro and ex vivo genome editing—such as gene delivery into fertilized eggs, chimeric antigen receptor (CAR)-T cells, or mesenchymal stem cells (MSCs)—can be achieved using established techniques including lipofection (cationic lipid-mediated transfection), electroporation (electric pulse-mediated delivery), viral vector systems, or microinjection [48]. To address the limitations of in vivo gene editing, CRISPR/Cas systems delivered via nanocarriers offer a promising solution. As a strategy for delivering enzymatically unstable substances such as nucleic acids and peptides, Toshihiko Tashima reviewed non-invasive transmembrane delivery methods for CRISPR/Cas9 ribonucleoproteins (Cas9 RNPs) into cells [49].

(9) Javiera Carrasco-Rojas et al. developed a nanostructured lipid carrier (NLC)-based delivery system for rifampicin. The NLCs exhibited sustained drug release, best described by the Korsmeyer–Peppas model, and significantly enhanced antibacterial activity against *Staphylococcus aureus*, with an IC_50_ value of 0.46 ng/mL—approximately three times lower than that of rifampicin alone [50]. The Korsmeyer–Peppas model is commonly used to analyze the drug release kinetics from pharmaceutical formulations [51,52,53].

Among various nanocarriers, nanogels are attracting increasing attention as a highly promising drug delivery platform. They are composed of hydrophilic cross-linked polymers capable of absorbing large amounts of water or biological fluids, along with encapsulated cargo materials. This structure imparts several advantageous properties, including high drug loading capacity, tunable permeability, controllable particle size, minimal immunogenicity, colloidal stability, biocompatibility, and biodegradability [54].

(10) Tapinarof is an aryl hydrocarbon receptor (AhR) agonist that exerts therapeutic effects by downregulating proinflammatory cytokines, and is used in the treatment of psoriasis [55]. Clinical studies have shown that tapinarof cream (1%) is both effective and generally well-tolerated [56]. Barbara Balogh et al. explored emerging therapeutic strategies involving nanogel-based formulations of tapinarof as a novel alternative for topical psoriasis treatment. Nanogels offer several key advantages in transdermal drug delivery, including enhanced skin hydration, targeted delivery to skin layers, and controlled drug release. In their study, nanogel formulations containing tapinarof were prepared using Carbopol 940 and 936 polymers, along with excipients such as Tween 80, Kolliphor, and oleic acid, which facilitated the release of tapinarof [57].

(11) Moreover, Shaul D. Cemal et al. demonstrated that voriconazole-loaded nanohydrogels exhibited potent antifungal activity against clinical fungal isolates obtained from the respiratory secretions of cystic fibrosis (CF) patients. Drug release was assessed by detecting the fluorescence of the released voriconazole. These findings provide a foundation for future in vivo investigations [58].

Overall, the articles in this Special Issue highlight recent advances in nanoparticle-mediated targeted drug delivery systems and are expected to contribute significantly to the development of this field. We extend our sincere gratitude to all the authors for their outstanding contributions, as well as to the Assistant Editors for their invaluable support.
